# The clinical and biological significance of HER2 over-expression in breast ductal carcinoma in situ: a large study from a single institution

**DOI:** 10.1038/s41416-019-0436-3

**Published:** 2019-05-08

**Authors:** Islam M. Miligy, Michael S. Toss, Kylie L. Gorringe, Andrew H. S. Lee, Ian O. Ellis, Andrew R. Green, Emad A. Rakha

**Affiliations:** 1 Nottingham Breast Cancer Research Centre, Division of Cancer and Stem Cells, School of Medicine, Nottingham City Hospital, The University of Nottingham, Nottingham, UK; 2 Histopathology Department, Faculty of Medicine, Menoufia University, Menoufia, Egypt; 3 Histopathology Department, South Egypt Cancer Institute, Assiut University, Assiut, Egypt; 4 Cancer Genomics Program, Peter MacCallum Cancer Centre, Melbourne, University of Melbourne, Parkville, Australia; 5 The Sir Peter MacCallum Department of Oncology, University of Melbourne, Parkville, Australia

**Keywords:** Oncogenesis, Breast cancer

## Abstract

**Background:**

Previous studies have reported up to 50% of ductal carcinoma in situ (DCIS), is HER2 positive, but the frequency of HER2-positive invasive breast cancer (IBC) is lower. The aim of this study is to characterise HER2 status in DCIS and assess its prognostic value.

**Methods:**

HER2 status was evaluated in a large series of DCIS (*n* = 868), including pure DCIS and DCIS associated with IBC, prepared as tissue microarrays (TMAs). HER2 status was assessed using immunohistochemistry (IHC) and chromogenic in situ hybridisation (CISH).

**Results:**

In pure DCIS, HER2 protein was over-expressed in 9% of DCIS (3+), whereas 15% were HER2 equivocal (2+). Using *CISH*, the final HER2 status was positive in 20%. In mixed DCIS, *HER2* amplification of the DCIS component was detected in 15% with amplification in the invasive component of only 12%. HER2-positive DCIS was associated with features of aggressiveness (*p* < 0.0001) and more frequent local recurrence (*p* = 0.03). On multivariate analysis, combined HER2+/Ki67+ profile was an independent predictor of local recurrence (*p* = 0.006).

**Conclusions:**

The frequency of HER2 positivity in DCIS is comparable to IBC- and HER2-positive DCIS is associated with features of poor prognosis. The majority of HER2 over-expression in DCIS is driven by gene amplification.

## Background

Ductal carcinoma in situ (DCIS) of the breast was less common before the 1980s; however, with the introduction of mammographic screening, the incidence has increased dramatically and now comprise approximately 20% of breast cancers.^1^ Over the last decade, breast carcinoma in situ incidence rates have increased by almost half (46%) in the UK (http://www.cancerresearchuk.org/health-professional/cancer-statistics). The mortality from DCIS is very low, with a maximum of 3% at 20 years of follow-up and the survival after surgery is excellent.^2^

Following a diagnosis of DCIS, women are at elevated risk for disease progression,^3^ but not all DCIS cases progress to invasive carcinoma if untreated with an estimated risk in the range of 25–50%.^4^ Therefore, the main aim of DCIS management is to prevent progression and local recurrence, particularly invasive recurrence.^5^

Clinicians are unable to precisely predict the risk of local recurrence or progression to invasive cancer in patients with DCIS following their treatment, a major concern that needs to be addressed. Treatment is by mastectomy, or lumpectomy with or without adjuvant radiotherapy.^6^ Factors such as young patient age, large tumour size,^7^ positive margins, comedo necrosis and high-nuclear grade^8^ are associated with a higher risk of recurrence with some emerging data suggesting that *ERBB2*-amplified (HER2) DCIS could present a higher risk of recurrence.^9^ The Van Nuys Prognostic Index is a popular risk assessment tool combining patient age, lesion size, nuclear grade and margin status for treatment decisions.^10^

HER2 is an established negative prognostic factor in invasive breast cancer.^11^ The prognostic significance of HER2 status in DCIS is, however, less clear.^1,12^ The relationship of HER2 to risk of recurrence and its role in the progression from in situ to invasive cancer have been debated.^9^ HER2 over-expression is reported to be more frequent in DCIS than in invasive cancer.^13^ This may seem contradictory, as HER2 is proposed to play a role in tumour progression. Some studies report an even higher proportion of HER2 positivity in microinvasive cancer^14^ and, in preoperative tumour biopsies displaying DCIS, HER2 over-expression has been related to increased incidence of invasive carcinoma in the surgical specimen.^15^ Furthermore, HER2 positivity is associated with high-histopathological grade both in invasive cancer and in DCIS.^16^

Immunohistochemical staining (IHC) can be used as a surrogate marker of gene-expression profiling in invasive breast cancer, utilising ER, PR, HER2 and Ki-67 protein expression to split patients into four different molecular phenotypes.^17^ Similar subtypes have been found in DCIS, but the prognostic significance of grouping DCIS into these subtypes is not yet clear. Some studies have suggested the most appropriate treatment could be identified by evaluating individual expression of genes or receptors in DCIS.^18^

IHC staining is the predominant method of determining HER2 status in breast cancer specimens due to its relative ease to perform with rapid turnaround time and lower cost.^19^ Although commercially available antibodies have shown wide variation in sensitivity and specificity for formalin-fixed paraffin-embedded (FFPE) tissue samples, in which tissue fixation and pre-treatment have a substantial effect on staining,^20,21^ a significant improvement of the diagnostic standards in clinical histopathology with quality control measures were able to improve diagnostic performance in clinical practice.^22^

*HER2* gene amplification is primarily detected by in situ hybridisation and uses fluorescence (FISH) to detect the signals. This method is expensive and requires expertise with fluorescent microscopy using appropriate filters.^23^ Chromogenic in situ hybridisation (CISH) offers an alternative method, and whilst it utilises in situ hybridisation technology of FISH, it takes advantage of a chromogenic signal detection that is assessed using light microscopy and therefore costs much less than FISH. CISH is able to detect *HER2* gene amplification and to minimise, if not eliminate, the borderline category with IHC.^24^ The performance of CISH in *HER2* testing has rarely been tested in series, so we aimed to characterise HER2 status using IHC and CISH in DCIS and assess its prognostic value in a large and well annotated cohort.

## Methods

### Study cohort and tissue microarray

This retrospective study was conducted on a consecutive series of primary pure DCIS cases diagnosed, and treated, between 1990 and 2012 at Nottingham City Hospital, Nottingham, UK. Exclusion of referral, miscoded and recurrent cases resulted in 776 cases of pure primary DCIS with available (FFPE tumour blocks for TMA construction. During the same time-period, a series of 239 cases diagnosed as synchronous DCIS and invasive tumours (mixed DCIS and invasive breast cancer (IBC)) was also collected for comparison. Patients’ demographic information, histopathological parameters, surgical management, radiotherapy and patient outcome data were collated. Tumour size was the sum of the primary excision and the re-excision tumour size (mm). Tumour grade was classified according to the three-tier nuclear grading system; low, intermediate and high-nuclear grades.^25^ Local recurrence free survival was calculated based on the time (in months) from the date of primary surgical treatment to the time of ipsilateral local recurrence. Patients who developed contralateral disease following DCIS diagnosis were censored at the time of diagnosis of the contralateral cancer.

The median follow-up period in the pure DCIS cohort treated with breast conserving surgery was 118 months (range: 2–240 months), during which 75 patients (12%) developed ipsilateral local recurrence, including invasive carcinoma in 48 (62%). Of the 308 patients treated with breast conserving surgery alone, there were 67 recurrences (22%), of which 38 (57%) cases were invasive recurrences. Amongst the 93 cases treated with breast conserving surgery and radiotherapy, there were 8 recurrences (9%) (2 invasive and 6 DCIS).

Tissue microarrays (TMAs) were prepared from representative DCIS lesions of the pure cases and from DCIS and invasive tumours from the mixed cases as previously described.^26^ The TMA was constructed using 3D Histech^®^ Grand Master^®^, Budapest, Hungary, whereby representative cores of 1 and 0.6 mm from appropriate viable tumour area were taken from DCIS and invasive tumour samples, respectively, avoiding scanty, single focus, necrotic tumour zones and thinner tumour blocks than 4 mm. Each case was represented by a single-core biopsy; however, cases with more than one histologic tumour type were represented by more than one core.

### Immunohistochemistry and chromogenic in situ hybridisation

The expression of ER, PR, HER2 and Ki67 was evaluated using IHC on the TMA sections. Positive and negative controls were included in every assay (Supplementary material). MIB1 antibody was used for evaluation of Ki67 expression. Nuclear staining was scored for ER, PR and Ki67 and membrane staining for HER2. HER2 status was assessed using the HercepTest scoring method^27^ where 0 (no membrane staining or incomplete staining of <10% of cells), 1+ (weak or moderate incomplete membrane staining of >10% cells), 2+ (strong, complete membrane staining in ≤10% of tumour cells or weak/moderate complete membrane staining in ≥10% of tumour cells) or 3+ (strong, complete membrane staining in > 10% of tumour cells). HER2 status was considered negative if the immunohistochemical score was 0 or 1+, equivocal if the score was 2+ and positive if the score was 3+.

In equivocal cases, *HER2* gene amplification was determined by CISH. This was performed using the ZytoDot 2C SPEC ERBB2/CEN 17 Probe Kit, Germany. Interpretation of CISH was performed using a Nikon microscope equipped with a ×63 objective lens. At least 20 malignant, nonoverlapping cell nuclei were scored to assess *HER*2 gene copy number. Chromosome 17 (represented by the red signals) was used as an internal control particularly in the low *HER*2 gene copy number cases as per the recommended protocol*. HER*2 gene amplification was defined as six or more signals per nucleus or when clusters (clumps of aggregated green signals) were identified in the cell nuclei in more than 50% of tumour cells.^28^ If the average copy number was ≥4 to <6 per nucleus (equivocal), another 20 tumour cells were enumerated, and the final average copy number of the case was calculated from the total of 40 cells. In cases with multiple cores (*n* = 26), HER2 score was initially obtained by IHC followed by confirmation with CISH to reach the final status of the case (Supplementary Table 1).

For ER and PR, a 1% cut-off value was used to dichotomise cases into positive and negative.^29^ For Ki67 the cut-off for differentiating low- and high-proliferative groups was 14%.^30^

### Statistical analysis

Statistical analyses were performed using SPSS v24 (Chicago, IL, USA) for windows. Association between final HER2 status and clinicopathological parameters using categorised data in the pure DCIS cohort was evaluated using chi-squared test. Survival rates were determined using the Kaplan–Meier method and compared by the log-rank test. Multivariate survival analyses using Cox proportional hazard regression model was used to evaluate the associations between independent variables and local recurrence. Multivariable hazards ratio and their corresponding 95% confidence intervals (CI) were initially performed for each marker alone and then for the combination of biomarkers. All tests were two-tailed and a *p* value of less than 0.05 was considered as statistically significant.

## Results

A total of 651 cases of pure DCIS patients (Supplementary Table 2) and 217 cases mixed with invasive carcinoma were suitable for analysis. When pure DCIS was compared to DCIS mixed with invasive disease, mixed DCIS was of higher nuclear grade with comedo necrosis (*p* < 0.0001).

### Assessment of HER2 status

**Fig. 1:**
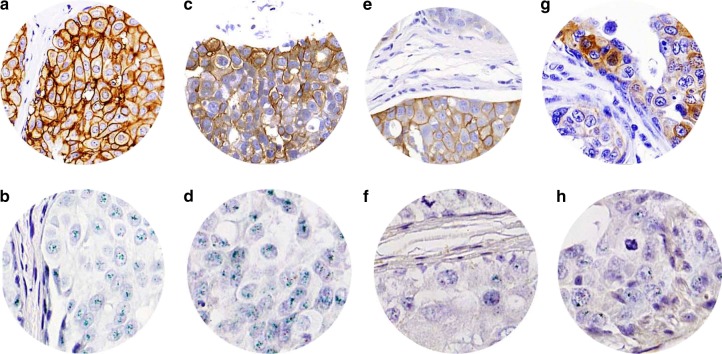
DCIS showing HER2 3+ (**a**) confirmed by CISH (**b**), HER2 2+ (**c**) showing gene amplification (**d**), HER2 2+ (**e**) without amplified gene (**f**) and HER2 1+ (**g**) showing no gene amplification (**h**)

**Fig. 2:**
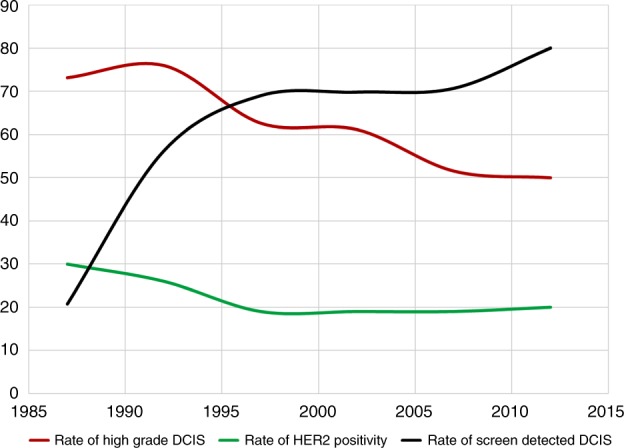
The rate of HER2 positivity, grade and screen detected DCIS change over time. The graph shows that the rate of high grade DCIS together with HER2 positivity is declining over years in contrast to the rate of screen detected DCIS

In the pure DCIS group, 20% of cases were HER2 positive. Immunohistochemistry for HER2 was scored as 3+ in 9%, 2+ in 15% and 0 or 1+ in 76% (Table 1). All 3+ cases showed *HER2* amplification with CISH. Totally, 73% of 2+ cases showed *HER2* amplification (4 cases in which CISH could not be assessed due to technical issues and were excluded) (Fig. 1). CISH confirmed high-copy number of *HER2* gene in all IHC 3+ cases. Of the 493 cases with 0/1+ score using IHC, only one case showed *HER2* amplification. When IHC results were assessed with the exclusion of equivocal (2+) tumours, agreement between IHC and CISH in 0, 1+ and 3+ cases was 99.7% (*κ*-coefficient = 0.997) (Table 1 and Supplementary Table 3). Interestingly, when the overall rate of positivity was analysed over the period of the study, the frequency of positivity reduced from 30% in the 1990s to 20% in 2012 (Fig. 2).

**Table 1:** HER2 status in pure DCIS cases using IHC and CISH

CISH	HER2 IHC in pure DCIS			
	0/1+ (*n* = 493) (%)	2+ (*n* = 98) (%)	3+ (*n* = 56) (%)	Total (*n* = 647) (%)
Amplified	1 (0.2)	72 (73.5)	56 (100.0)	129 (20.0)
Non-amplified	492 (99.8)	26 (26.5)	0 (0.0)	518 (80.0)

*DCIS* ductal carcinoma in situ, *IHC* immunohistochemistry, *CISH* chromogenic in situ hybridisation

Twenty-six cases had multiple cores across the TMAs, of which 15 were concordant for IHC and CISH (13 for IHC 0/1+ and 2 for IHC 2+). Of the 11 cases with discordant IHC, which included all possible pairwise combinations of 0/1+, 2+ and 3+, the CISH results were consistent across the cores, indicating a greater reliability of CISH over IHC in detecting true amplification particularly in heterogeneous cases as well as in limited samples such as core biopsies.

In mixed DCIS and invasive carcinoma cases, the DCIS component was HER2 positive in 15% and the invasive component in 12% (*p* = 0.051, Table 2 and Supplementary Tables 4–7). All eight discordant cases had extensive DCIS; six of them were of high grade and ER positive.

**Table 2:** HER2 status in DCIS cases mixed with invasion

(A) HER2 status in DCIS cases mixed with invasion using IHC and CISH								
CISH	HER2 IHC DCIS component				HER2 IHC invasive component			
	1+ (*n* = 185) (%)	2+ (*n* = 10) (%)	3+ (*n* = 22) (%)	Total (*n* = 217) (%)	1+ (*n* = 192) (%)	2+ (*n* = 2) (%)	3+ (*n* = 23) (%)	Total (*n* = 217) (%)
Amplified	5 (2.7)	5 (50.0)	22 (100.0)	32 (14.7)	1 (0.5)	1 (50.0)	23 (100.0)	25 (11.5)
Non-amplified	180 (97.3)	5 (50.0)	0 (0.0)	185 (85.3)	191 (99.5)	1 (50.0)	0 (0.0)	192 (88.5)

(B) Final HER2 status of DCIS and invasive component of DCIS cases mixed with invasion			
	Invasive HER2		Total no. (%)
DCIS HER2	Positive	Negative	
Negative	1	184	185 (85.3)
Positive	24	8	32 (14.7)
Total	25 (11.5)	192 (88.5)	217

*DCIS* ductal carcinoma in situ, *IHC* immunohistochemistry, *CISH* chromogenic in situ hybridisation

### Correlation with clinicopathological parameters and molecular biomarkers

In pure DCIS, HER2 positivity was associated with larger tumour size, high-nuclear grade, comedo type DCIS, negative hormone receptor status, high-Ki67 proliferation index and abnormal expression of p53 (all *p* < 0.0001). HER2-positive cases were more frequent in patients treated by mastectomy (*p* = 0.001) (Table 3).

**Table 3:** Association between final HER2 status and the clinico-pathological parameters of pure DCIS cohort

Parameter	HER2 status			
	Total (*n* = 646) *n* (%)	Positive (*n* = 128) *n* (%)	Negative (*n* = 518) *n* (%)	*χ*^2^ (*p* value)
Age (years)*				
Less than 40	22 (3.4)	7 (6)	15 (3)	2.12
Between 40 and 60	376 (58.2)	74 (58)	302 (58)	(0.347)
More than 60	248 (38.4)	47 (36)	201 (39)	
Presentation				
Screening	336 (52)	69 (54)	267 (51)	0.23
Symptomatic	310 (48)	59 (46)	251 (49)	(0.632)
DCIS size (mm)				
Less than 16	223 (34.8)	27 (22)	196 (38)	20.63
Between 16 and 40	247 (38.5)	47 (37)	200 (39)	(**<0.0001**)
More than 40	171 (26.7)	52 (41)	119 (23)	
Nuclear grade				
Low	87 (13.5)	0 (0)	87 (17)	58.58
Intermediate	166 (25.7)	13 (10)	153 (29)	**(<0.0001)**
High	393 (60.8)	115 (90)	278 (54)	
Comedo necrosis				
Yes	411 (63.8)	111 (87)	300 (58)	36.28
No	233 (36.2)	17 (13)	216 (42)	**(<0.0001)**
Management				
Mastectomy	339 (52.5)	84 (66)	255 (49)	11.06
Breast conserving surgery	307 (47.5)	44 (34)	263 (51)	(**0.001**)
Radiotherapy				
Yes	95 (14.7)	19 (15)	76 (15)	0.002
No	551 (85.3)	109 (85)	442 (85)	(0.961)
Oestrogen receptor status				
Positive	426 (75.3)	45 (40)	381 (84)	88.86
Negative	140 (24.7)	66 (60)	74 (16)	**(<0.0001)**
Progesterone receptor status				
Positive	302 (46.7)	18 (14)	284 (55)	68.51
Negative	344 (53.3)	110 (86)	234 (45)	**(<0.0001)**
Ki67 index				
High proliferative	108 (23.0)	64 (59)	97 (27)	37.46
Low proliferative	361 (77.0)	45 (41)	263 (73)	**(<0.0001)**

*p* value in bold: significant

*DCIS* Ductal carcinoma in situ, *n* number

*Age: categorised according to the Van Nuys Prognostic Index (VNPI)

### Associations with outcome

Nuclear grade, comedo necrosis and margin width were individually associated with the development of all ipsilateral recurrences (*p* = 0.023, 0.013 and 0.029, respectively). When recurrences were stratified into in situ and invasive recurrences, HER2 was associated with the development of DCIS local recurrence. Patients with HER2+/Ki67-high DCIS had a high likelihood of developing invasive local recurrence (*p* = 0.004). On multivariable analysis, patients with HER2+/Ki67-high DCIS had a hazard ratio of 2.5 (95% CI: 1.7–55.0, *p* = 0.006), compared to women with other profiles (Table 4).

**Table 4:** Univariate and multivariate analysis

(A) Univariate analysis for predictors of local recurrence in pure DCIS patients treated with breast conserving surgery		
Outcome	HR (95% CI)	*p* Value*
All local recurrence		
Tumour size	1.31 (0.36–2.55)	0.098
High tumour grade	3.43 (1.18–9.96)	**0.023**
Radiotherapy	0.95 (0.47–1.91)	0.890
ER status	1.09 (0.58–2.05)	0.777
HER2 status	0.89 (0.45–1.74)	0.734
Ki 67 status	2.61 (0.33–5.01)	0.089
Comedo necrosis	3.63 (1.44–7.82)	**0.013**
Margin width	0.63 (0.34–0.91)	**0.029**
DCIS local recurrence		
Tumour size	1.01 (0.46–2.23)	0.423
High tumour grade	1.90 (0.21–4.72)	0.569
Comedo necrosis	1.24 (0.73–1.99)	0.372
Margin width	1.83 (0.15–11.96)	0.180
Radiotherapy	0.75 (0.35–1.59)	0.459
ER status	0.76 (0.29–1.92)	0.551
HER2 status	2.51 (2.11–11.45)	**0.030**
Ki 67 status	2.44 (0.13–2.29)	0.067
Invasive local recurrence		
Tumour size	2.63 (1.13–6.09)	**0.024**
High tumour grade	1.15 (0.25–5.24)	0.871
Comedo necrosis	1.49 (0.814–2.76)	0.194
Margin width	0.69 (0.43–2.47)	0.578
Radiotherapy	1.17 (0.35–3.86)	0.780
ER status	1.01 (0.45–2.65)	0.833
HER2 status	0.49 (0.17–1.41)	0.184
Ki 67 status	1.28 (0.41–3.88)	0.668

(B) Multivariate Cox proportional hazards for factors associated with local recurrence in pure ductal carcinoma in situ patients		
Variable	HR (95% CI)	*p* Value
Ductal carcinoma in situ size	0.62 (0.31–3.47)	0.311
Ductal carcinoma in situ grade	2.21 (1.15–70.85)	**0.031**
Comedo necrosis	0.32 (0.12–0.87)	**0.026**
Margin width	0.89 (0.11–7.46)	0.911
HER2+/Ki67-high group	1.52 (1.64–60.74)	**0.001**

*DCIS* ductal carcinoma in situ, *HR* hazard ratio, *CI* confidence interval, *ER* oestrogen receptor

*p Values are corrected according to Bonferroni multiple testing correction; bold facing is significant

## Discussion

HER2 status can be detected by analysing the number of gene copies or measuring the amount of protein expression. The most widely used methods at present are IHC, FISH and CISH due to their propensity in evaluating HER2 in FFPE tissues in invasive carcinoma.^31^ Most HER2 studies have been performed by IHC, which is widely accessible, easy to perform at a reasonable cost and is appropriate for initial HER2 assessment.^32^ However, in cases with an IHC score of 2+, the inter-observer agreement is poor, and the predictive value is unsatisfactory for clinical use. This led to the recommendation of additional testing measuring *HER*2 gene copy number status using an in situ hybridisation technique such as FISH or CISH to avoid inaccurate prognostication and inappropriate treatment.^32,33^

Although FISH is a fairly objective and quantitative procedure in detecting *HER2* gene amplification, it has many drawbacks, including its cost, the essential need for a fluorescence microscope, its temporary signal which requires a special camera and is technically challenging with TMA preparations.^34^ CISH is an alternative in situ hybridisation method to analyse gene amplification.^35^ It requires an ordinary bright field microscope and the method is less cumbersome and more economical with permanent signal intensity. Pathologists are able to correlate findings with the underlying tumour histo-morphology. Furthermore, several studies have demonstrated good correlation between CISH and FISH results.^36,37^ CISH is either performed as dual colour or single colour with an advantage of the latter over the former in being easy to count signals especially in amplified cases.^38^

To the best of our knowledge, this is the first study to assess HER2 in DCIS using in situ hybridisation. In this study, similar to a previous study, but in invasive tumour,^31^ the concordance between 3+ IHC and CISH-amplified cases was 100%, denoting all HER2 over-expressing cases as having *HER2* gene amplification. In contrast, the proportion of 2+ IHC that were *HER2* amplified, as detected by CISH, was 73.5%, which is lower than previously reported (93%).^39^ The 26.5% (26/98) IHC-equivocal/*HER2* non-amplified tumours in this study, all of which had 2+ IHC scores, is higher than the result obtained by other studies on invasive tumours.^40–42^ The concordance between 0/1+ IHC and *HER2* non-amplified tumours was 99.7%. A similar finding was reported by Zhao and colleagues and they considered that there is a small undetermined percentage of amplified *HER2* without protein over-expression.^40^ The same study and other FISH studies on invasive carcinoma also reported rare 3+ IHC tumours that were non-amplified,^40–43^ however, we did not detect any such cases.

The low-amplified *HER2* category (≥4 to <6 CISH signals) was the most difficult to interpret, requiring an accurate enumeration of gene copy and addition of more tumour cells to assess the final status. This step was particularly important because these cases could either resolve as amplified (4/7) or non-amplified (3/7). Signal clustering, more probably a result of intra-chromosomal amplification of homogeneously staining regions, was immediately evident in highly amplified cases and was easily evaluated.

We observed a lower HER2 expression in DCIS compared to the previously published literature.^1,15^ This might be related to the increasing detection of low- and intermediate-grade DCIS by screening mammography. In support of this the HER2 positivity was higher (30%) in the earlier period of the study and declined over time to 20% (Fig. 2).

The question of whether or not TMA cores are representative of whole tumours is frequently raised, as some tumours are heterogeneous and so a small sample of tissue may not always display the same biological characteristics as a larger section.^34^ To increase the credibility of the TMA technique while reducing the probability of error associated with the difficulty of obtaining a representative sample, some studies use multiple cores from the same donor tissues as it has been reported that only a few (up to four) cores are required to achieve a 100% agreement.^44^ However, even when as many as ten tissue cores are taken from a tumour, some disagreement may be noted.^45,46^ In the current study, 1 mm diameter cores were used to construct the TMA of the pure DCIS tumours, increasing the probability of accurate sampling by taking large TMA cores. In cases with multiple cores, the initial HER2 status was assessed at the protein level followed by confirmation with in situ hybridisation. The high agreement rate between IHC and CISH results suggests that it is possible to apply TMAs to DCIS research, with the caveat that, for HER2, the CISH results may be more reliable, countering the discrepancy that can be observed in IHC for either technical or biological reasons.

Our subsequent goal in this study was to assess the role of HER2 in progression of DCIS. HER2-positive DCIS was associated with predictors of local recurrence like larger tumour size, high-nuclear grade, comedo type DCIS, negative-hormone receptor status and high-Ki67 proliferation index, which was consistent with other studies.^12,47–51^ Local recurrence (invasive or DCIS) was associated with higher-grade, comedo necrosis and margin status. DCIS local recurrence was associated with HER2 positivity and invasive local recurrence was associated with tumour size. Similar to some studies, HER2 positive tumours were associated with DCIS local recurrence.^52,53^

DCIS patients with *HER2* amplification had higher nuclear grade lesions and therefore are probably at risk of relapse more frequently than the *HER2* unamplified group, but the prognostic role of HER2 over-expression in DCIS is still not fully clarified. Some studies suggest that patients with *HER2* amplified DCIS are more frequently high-nuclear grade and this aspect is related to an increased risk of relapse.^54,55^ In fact, some studies suggest that HER2 plays a major role in the transition from DCIS to IDC,^18,56^ while others do not support this.^9,57^ In the current study, *HER2* amplification was more frequent in the pure DCIS cohort (20%) than in the mixed DCIS (15%) despite the more prevalent higher-grade DCIS in the mixed cases. HER2 positivity alone was not predictive of recurrence as a whole or as invasive disease but showed a trend to DCIS recurrence. This result is consistent with our observation that HER2 positive DCIS tend to extend within the ductal system and even in the epidermis (Paget’s disease) than associated with invasive disease. It is our personal observation that when HER2-positive DCIS is associated with early invasion, that invasive disease may show aggressive features. Currently, HER2-positive invasive tumours are treated more aggressively regardless of its size.^58^ This may suggest that HER2-positive DCIS tumour cells although highly proliferative are not highly invasive, whilst those that acquire invasive properties became aggressive and can metastasise.

In order to improve the risk stratification and therefore treatment recommendations, there is a need to identify potential predictors of invasive and/or non-invasive recurrence following conserving breast surgery. HER2/Ki67 positivity was a predictor of recurrence, independent of other studied clinicopathological parameters such as nuclear grade and presence of comedo necrosis. In contrast to a previous study,^59^ the combination of ER status with HER2 and Ki67 further added additional predictive information. This difference may be due to the differences in patient populations, or to differences in the coding of HER2 positivity. The previous study coded DCIS lesions with equivocal immunostaining for HER2 (score of 2+) as positive. We performed in situ hybridisation on all equivocal cases and only lesions with amplification were coded as HER2 positive.

In the present study, HER2 expression in the invasive component was seen less frequently as compared to the DCIS component adjacent to invasive carcinoma and pure DCIS although this was of borderline statistically significant. Similarly, other studies^54,60^ have observed that HER2 positivity is often in patients with pure DCIS compared to those with microinvasive or invasive carcinoma. As it has been reported that patients with HER2+ invasive cancer have a poorer prognosis than those whose tumours do not express HER2,^61^ the higher incidence of expression found in DCIS may therefore seem paradoxical. It was postulated that HER2 expression happens during the process of atypical hyperplasia in DCIS, and loss of HER2 expression occurs as DCIS develops into invasive disease. Another hypothesis is that HER2 over-expression in invasive carcinoma does not develop from DCIS but from the associated atypical hyperplasia.^62,63^ Another theory explaining this phenomenon is that most invasive cancers develop from DCIS tumours, which have low expression of HER2 but are highly proliferative.^54^ Because such tumours progress rapidly, they are in the DCIS stage for only a short period and would therefore be under-represented in population samples.^54^

In summary, CISH is a promising, practical test that can be used in conjunction with IHC to determine HER2 status in DCIS. IHC is easy to perform, relatively inexpensive, and able to detect a majority of breast cancer patients whose tumours have negative (0 or 1+) or positive (3+) HER2 status. This IHC/CISH test stratification not only identifies the IHC borderline cases, but also keeps testing costs for HER2 status at a reasonable minimum. As HER2 status is not currently routinely measured in clinical practice, we aimed to show statistically significant correlations between the development of local recurrence as well as poor prognostic pathologic factors and HER2 positive DCIS which could alert clinicians. Our results might suggest considering routine assessment of HER2 in DCIS, similar to invasive breast cancer.

We also confirmed the suitability of the TMA technique for assessment of *HER2* gene amplification status in tumours arising from different patients, while maintaining a representative tissue sample in each case, with good reproducibility and credible results.

To conclude, the frequency of HER2 positivity, driven by gene amplification, in DCIS is comparable to IBC and in combination with Ki67, is an independent predictor of recurrence. Our results suggest the consideration of routine assessment of HER2 status for DCIS, as it is commonly done for IBC.

### Limitations of the study

This study has been conducted on TMA sections, which might underestimate the role of tumour heterogeneity. However, all cases in our cohort were carefully reviewed before TMA construction and used multiple cores for cases with heterogeneous morphology. The lower number of radiotherapy-treated cases after BCS was due to the prevalence of mastectomy in the earlier period of the study; which constituted the majority of cases.

## Supplementary information

Supplementary Material Including Supplementary Tables 1-7

## Data Availability

The authors confirm the data that has been used in this work is available on reasonable request.
