# Supplementary material for: The clinical and biological significance of HER2 over-expression in breast ductal carcinoma in situ: a large study from a single institution
Source: Br J Cancer. 2019 May 8;120(11):1075–82. doi: 10.1038/s41416-019-0436-3 (PMC6738110; doi:10.1038/s41416-019-0436-3)
Supplement: Supplementary file 1 — Supplementary Material Including Supplementary Tables 1-7 [file 41416_2019_436_MOESM1_ESM.docx]

**Supplementary Material**: IHC protocol for the antibodies ER, PR, HER2, Ki 67 and CISH.

As regard to HER2 IHC, sections were stained using Novocastra Novolink polymer detection system (Leica, Newcastle, UK). 4µm TMA sections were deparaffinised on thermal plate then rehydrated on Leica autostainer (2x xylene, each 5 minutes, 2x of 100% ethanol at 2 minutes each, 2x of 95% ethanol at 2 minutes each, 2x of 70% ethanol at 2 minutes each and deionised water, dH2O for 2 minutes). Heat induced epitope retrieval using citrate buffer was performed for all antibodies except for HER2 where no antigen retrieval was performed. The sections were incubated with peroxidase block for 5 minutes. After 3x washes with Tris Buffered Solution – Tween® 20 (TBS-Tween® 20), application of protein block was done for 5 minutes. After 3x washes, incubation of the sections with polyclonal rabbit anti c-erb B2 (1:400) for 30 minutes, monoclonal mouse anti-PR (clone PgR636, 1:150) for 30 minutes and Ki 67 (clone MILB1, 1:100) for 60 minutes at room temperature (RT) was done. Washing as previously described was done followed by application of post-primary antibody for 30 minutes. After 3x washes, incubation with Novolink Polymer for 30 minutes. 3,30-Diaminobenzidine tetrahydrochloride (Novolink DAB substrate buffer) was used as a chromogenic substance after 3x washes. Sections were counterstained with haematoxylin for 6 minutes and then dehydrated in Leica autostainer then cover-slipped with Distyrene Polystyrene Xylene (DPX). As regard to ER, the sections were stained on the Ventana Benchmark® ULTRA system (Tucson, Arizona, USA) using Ventana anti-ER (SP1) Rabbit Monoclonal Primary Antibody as per the recommended protocol. Sections were deparaffinised, and antigen retrieval was performed with Cell Conditioner 1 (CC1) for 60 min. Then the primary antibody was applied for 16 minutes at 37°C followed by the OptiView HQ Linker for 8 minutes and the OptiView HRP Multimer for 8 minutes. Counter-staining was performed with Mayer’s haematoxylin.

CISH: Slides bearing TMA sections of pure DCIS tumours as well as cases associated with invasion were deparaffinised and incubated for 10 minutes (at 70°C on thermal plate) followed by dewaxing in xylene (twice, each is 5 minutes), and then gradient ethanol (each 5 minutes). Incubation of the slides with hydrogen peroxide, H_2_O_2_ (3%, for 5 minutes), followed by washing in distilled water (D.W.) was done. Treatment of the slides by pre-heated pre-treatment solution EDTA (PT2) for 15 minutes, was performed, followed by immediate transfer into distilled water (twice, 2 minutes each). Enzymatic digestion was carried out by drop-wise application of pepsin solution (ES1) to the tissue sections and incubation for 5 minutes at room temperature (RT) in a humidity chamber. After a wash in D.W, dehydration with graded ethanol followed by air dry sections was done. A ready to use DNA probe (ZytoDot 2C SPEC ERBB2/CEN 17 Probe, PD12) was applied to the slides (10μl per slide) and cover-slipped (22x22mm) then sealed with a layer of rubber cement. The sections were denatured on a thermal plate (80°C for 5 minutes), and hybridisation was carried out overnight at 37°C in a humidity chamber. After hybridisation, removal of the cover slip was done, and the slides were washed twice (each 5 minutes) with wash buffer SSC (WB1) (1^st^ wash at RT, and the 2^nd^ heated to 75°C). After two washes in D.W (1 minute each), immersion of the slides into 1x Wash Buffer TBS (prepared using WB5) was done. The *HER2* probe was detected by sequential incubation with anti-digoxigenin DIG/DNP-Mix (AB14) for 15 minutes (at 37°C in a humidity chamber), peroxidase polymer horseradish peroxidase HRP/AP-Polymer-Mix (AB13) for 15 minutes (at 37°C in a humidity chamber), AP-Red Solution B (SB6b) for 15 minutes (at RT) and HRP-Green Solution A (SB7a) for 15 minutes (at RT). Counter-staining by Nuclear Blue Solution (CS2) for 2 minutes was done followed by washing and dehydration in absolute ethanol followed by xylene. Covering the slides (22x22mm cover slips) using Alcoholic Mounting Solution (MT4) and air-dry slides for 30 minutes was the final step.

**Supplementary Table 1:** Distribution of HER2 status using IHC and CISH in cases with multiple cores.

| Number of TMA cores/case | HER2 IHC/core | | | HER2 CISH score |
| --- | --- | --- | --- | --- |
|  | 0/1+ | 2+ | 3+ |  |
| 3 | 1 | - | 2 | >6 |
| 3 | - | 1 | 2 | >6 |
| 2 | - | 1 | 1 | >6 |
| 2 | - | 2 | - | >6 |
| 2 | - | 2 | - | <4 |
| 2 | 1 | 1 | - | <4 |
| 2 | 1 | - | 1 | >6 |
| 2 | 1 | 1 | - | <4 |
| 2 | - | 1 | 1 | >6 |
| 2 | - | 1 | 1 | >6 |
| 2 | - | 1 | 1 | >6 |
| 2 | - | 1 | 1 | >6 |
| 2 | 1 | 1 | - | >6 |
| 2 | 2 | - | - | <4 |
| 2 | 2 | - | - | <4 |
| 2 | 2 | - | - | <4 |
| 2 | 2 | - | - | <4 |
| 2 | 2 | - | - | <4 |
| 2 | 2 | - | - | <4 |
| 2 | 2 | - | - | <4 |
| 2 | 2 | - | - | <4 |
| 2 | 2 | - | - | <4 |
| 2 | 2 | - | - | <4 |
| 2 | 2 | - | - | <4 |
| 2 | 2 | - | - | <4 |
| 2 | 2 | - | - | <4 |

**Supplementary Table 2:** Clinicopathological characteristics of patients with pure DCIS in the study.

| **Parameter** | **Total (n=651)**  **n (%)** | **BCS (n=308)**  **n (%)** | **Mastectomy (n=343)**  **n (%)** | ***χ*^2^**  **(*p* value)** |
| --- | --- | --- | --- | --- |
| **Age, years***  **Less than 40**  **Between 40 and 60**  **More than 60** | 22 (3)  379 (58)  250 (39) | 5 (2)  179 (58)  124 (40) | 17 (5)  200 (58)  126 (37) | 5.86  (0.053) |
| **Presentation**  **Screening**  **Symptomatic** | 337 (52)  314 (48) | 187 (61)  121 (39) | 150 (44)  193 (56) | 18.74  (**<0.0001)** |
| **DCIS Size (mm)***  **Less than 16**  **Between 16 and 40**  **More than 40** | 224 (35)  247 (38)  175 (27) | 170 (56)  114 (37)  21 (7) | 54 (16)  133 (39)  154 (45) | 161.11  (**<0.0001**) |
| **Nuclear Grade**  **Low**  **Intermediate**  **High** | 87 (13)  167 (26)  397 (61) | 53 (17)  84 (27)  171 (56) | 34 (10)  83 (24)  226 (66) | 9.92  **(0.007)** |
| **Comedo necrosis**  **Yes**  **No** | 416 (64)  233 (36) | 179 (59)  127 (41) | 237 (69)  106 (31) | 7.89  **(0.005)** |
| **Radiotherapy**  **Yes**  **No** | 96 (15)  555 (85) | 93 (30)  215 (70) | 3 (1)  340 (99) | 110.97  **(<0.0001)** |
| **Recurrence**  **Yes**  **No** | 75 (12)  576 (88) | 67 (22)  241 (78) | 8 (3)  335 (97) | 52.92  **(<0.0001)** |
| **Oestrogen Receptor (ER) Status**  **Positive**  **Negative** | 428 (75)  142 (25) | 211 (80)  50 (20) | 217 (70)  92 (30) | 8.53  **(0.004)** |
| **Progesterone (PR) Receptor Status**  **Positive**  **Negative** | 302 (46)  349 (54) | 159 (52)  149 (48) | 143 (42)  200 (58) | 6.44  **(0.011)** |
| **Ki67 Index****  **High Proliferative**  **Low Proliferative** | 110 (23)  361 (77) | 46 (21)  169 (79) | 64 (25)  192 (75) | 0.848  (0.357) |

**P value in bold: Significant**

DCIS: Ductal carcinoma in situ, BCS: Breast conserving surgery, *χ*^2^: Chi square.

*Age and size: categorised according to the Van Nuys Prognostic Index (VNPI).

** Ki67 Index: according to the 14% cut-off value.

**Supplementary Table 3**: Comparison between HER2 status in pure DCIS cases using IHC and CISH

| **CISH** | **HER2 IHC in pure DCIS** | | | |
| --- | --- | --- | --- | --- |
|  | **0/1+** (n=493) (%) | **2+** (n=98) | **3+** (n=56) | **Total** (n=647) (%) |
| **<4** | 489 (99) | 26 (27) | 0 (0) | 515 (80) |
| **4 to 6** | 3 (0.6) | 3 (3) | 1 (2) | 7 (1) |
| **>6** | 1 (0.4) | 69 (70) | 55 (98) | 125 (19) |

**Supplementary Table 4:** HER2 status of DCIS cases mixed with invasion using IHC

|  | Invasive HER2 (IHC) | | | Total |
| --- | --- | --- | --- | --- |
| DCIS HER2 (IHC) | 0/1+ | 2+ | 3+ |  |
| 0/1+ | 183 | 1 | 1 | 185 (85.3) |
| 2+ | 7 | 0 | 3 | 10 (4.6) |
| 3+ | 2 | 1 | 19 | 22 (10.1) |
| Total | 192 (88.5) | 2 (0.9) | 23 (10.6) | 217 |

**Supplementary Table 5:** Comparison between HER2 status of the DCIS component in cases mixed with invasion using IHC and CISH

|  | **DCIS HER2 (CISH)** | | | **Total** |
| --- | --- | --- | --- | --- |
| **DCIS HER2 (IHC)** | <4 | 4 to 6 | >6 |  |
| 0/1+ | 179 | 1 | 5 | 185 (85.3) |
| 2+ | 5 | 0 | 5 | 10 (4.6) |
| 3+ | 0 | 0 | 22 | 22 (10.1) |
| **Total** | 184 (84.8) | 1 (0.5) | 32 (14.7) | 217 |

**Supplementary Table 6:** Comparison between HER2 status of the invasive component in DCIS cases mixed with invasion using IHC and CISH

|  | **Invasive HER2 (CISH)** | | | **Total** |
| --- | --- | --- | --- | --- |
| **Invasive HER2 (IHC)** | <4 | 4 to 6 | >6 |  |
| 0/1+ | 190 | 1 | 1 | 192 (88.5) |
| 2+ | 1 | 0 | 1 | 2 (0.9) |
| 3+ | 0 | 0 | 23 | 23 (10.6) |
| **Total** | 191 (88) | 1 (0.5) | 25 (11.5) | 217 |

**Supplementary table 7:** HER2 status of DCIS and invasive component in DCIS cases mixed with invasion using CISH

| **DCIS HER2 (CISH)** | **Invasive HER2 (CISH)** | | | **Total** |
| --- | --- | --- | --- | --- |
|  | <4 | 4 to 6 | >6 |  |
| <4 | 182 | 1 | 1 | 184 (84.8) |
| 4 To 6 | 1 | 0 | 0 | 1 (0.5) |
| >6 | 8 | 0 | 24 | 32 (14.7) |
| **Total** | 191 (88) | 1 (0.5) | 25 (11.5) | 217 |
